# Real world heart failure epidemiology and outcome: A population-based analysis of 88,195 patients

**DOI:** 10.1371/journal.pone.0172745

**Published:** 2017-02-24

**Authors:** Núria Farré, Emili Vela, Montse Clèries, Montse Bustins, Miguel Cainzos-Achirica, Cristina Enjuanes, Pedro Moliner, Sonia Ruiz, José María Verdú-Rotellar, Josep Comín-Colet

**Affiliations:** 1 Heart Failure Programme, Department of Cardiology, Hospital del Mar, Barcelona, Spain; 2 Heart Diseases Biomedical Research Group, IMIM (Hospital del Mar Medical Research Institute), Barcelona, Spain; 3 School of Medicine, Universitat Autònoma de Barcelona, Barcelona, Spain; 4 Analysis on Demand and Activity Division, Catalan Health Service, Barcelona, Spain; 5 Ciccarone Center for the Prevention of Heart Disease, Department of Cardiology, Johns Hopkins Medical Institutions, Baltimore, Maryland, United States of America; 6 Welch Center for Prevention, Epidemiology and Clinical Research, Johns Hopkins University, Baltimore, Maryland, United States of America; 7 Jordi Gol Primary Care Research Institute, Catalan Institute of Heath, Barcelona, Spain; 8 Heart Failure Program, Cardiology Department, University Hospital Bellvitge, Hospitalet de Llobregat, Barcelona, Spain; 9 School of Medicine, Department of Clinical Science, University of Barcelona, Hospitalet de Llobregat, Barcelona, Spain; 10 IDIBELL (Bellvitge Biomedical Research Institute), Hospitalet de Llobregat, Barcelona, Spain; Azienda Ospedaliero Universitaria Careggi, ITALY

## Abstract

**Background:**

Heart failure (HF) is frequent and its prevalence is increasing. We aimed to evaluate the epidemiologic features of HF patients, the 1-year follow-up outcomes and the independent predictors of those outcomes at a population level.

**Methods and results:**

Population-based longitudinal study including all prevalent HF cases in Catalonia (Spain) on December 31st, 2012. Patients were divided in 3 groups: patients without a previous HF hospitalization, patients with a remote (>1 year) HF hospitalization and patients with a recent (<1 year) HF admission. We analyzed 1year all-cause and HF hospitalizations, and all-cause mortality. Logistic regression was used to identify the independent predictors of each of those outcomes. A total of 88,195 patients were included. Mean age was 77 years, 55% were women. Comorbidities were frequent. Fourteen percent of patients had never been hospitalized, 71% had a remote HF hospitalization and 15% a recent hospitalization. At 1-year follow-up, all-cause and HF hospitalization were 53% and 8.8%, respectively. One-year all-cause mortality rate was 14%, and was higher in patients with a recent HF hospitalization (24%). The presence of diabetes mellitus, atrial fibrillation or chronic kidney disease was independently associated with all-cause and HF hospitalization and all-cause mortality. Hospital admissions and emergency department visits the previous year were also found to be independently associated with the three study outcomes.

**Conclusions:**

Outcomes are different depending on the HF population studied. Some comorbidity, an all-cause hospitalization or emergency department visit the previous year were associated with a worse outcome.

## Introduction

Heart failure (HF) is nowadays an important health problem. Not only is HF associated with a high use of resources and healthcare cost [1–3], but prevalence of heart failure is increasing due to better care and treatment of HF and to the aging of the population [4]. Epidemiology of HF is changing and shifting towards a higher prevalence of patients with HF with preserved ejection fraction [1]. However, since most of the information we know about HF is based on selected populations (patients after a HF hospitalization, followed-up in heart failure units, included in randomized controlled trials or with reduced ejection fraction), the real epidemiology of HF is currently not completely known. Moreover, the outcome of HF is grim. Mortality rate is high and hospitalizations are frequent and associated with worse outcomes [5]. The majority of studies that analyzed HF outcomes have focused on HF hospitalizations. However, in patients with HF, all-cause hospitalizations can affect up to 23–58% of the patients at 1-year follow-up [6–10] and non-cardiovascular hospitalizations are associated with risk of subsequent mortality similar to cardiovascular hospitalizations [11]. Despite the evidence that all-cause hospitalizations are detrimental in HF patients, few studies have analyzed whether the factors associated with all-cause and HF-hospitalizations are different in HF patients [12]. Furthermore, most of the studies that analyzed factors associated with mortality or hospitalizations have focused on 30-day readmission [13] or have been carried out in selected populations, i.e. patients with depressed ejection fraction, patients after a HF hospitalization or followed-up by cardiologists [11,14]. Little is known on mortality and hospitalization at a population level. The identification of factors associated to both all cause and HF hospitalization could help us to tailor the treatment and follow-up strategies in high-risk patients in order to improve their outcome and decrease expenditure associated with HF. Hence, the aim of this study was to analyze the epidemiology and outcome of patients with HF at a population level and to identify factors associated with mortality, HF and all-cause hospitalization at 1-year follow-up.

## Methods

### Data source, study design and study population

The design of this study has been previously reported [3]. Briefly, the study was performed in the region of Catalonia (Northeastern Spain). Local Health Department (Catsalut) provides public universal healthcare coverage to all residents and since 2011 collects detailed information on healthcare usage for the entire population of Catalonia (7,553,650 inhabitants in 2012) [15]. It includes information from the Minimum Basic Dataset for Healthcare Units registry which includes hospitalization, primary care, skilled nursing facilities and mental health network, information on pharmacy prescription and expenditure, and a registry on the billing record, which includes outpatient visits with specialists, emergency department visits, non-urgent medical transportation, ambulatory rehabilitation, domiciliary oxygen therapy and dialysis. The registry has an automated data validation system and external audits are performed periodically. Episodes of inpatient care attended in private health centers could not be captured because private hospitals do not use the Personal Health Identification Number. Nevertheless, use of private hospital is scarce for HF patients and the majority of unplanned HF hospitalizations (98%) are done in public hospitals.

The final study population comprised the 88,195 prevalent HF cases by December 31st 2012 who were 15 years or older. HF diagnosis was defined according to the International Classification of Diseases, Ninth Revision, Clinical Modification (ICD-9-CM) (see S1 File for Codes). Patients were divided in 3 groups: patients who had never been hospitalized due to HF, patients with a remote HF hospitalization (>1 year, i.e. before December 31st 2011) and patients with a recent (<1 year, between January 1st-December 31st 2012) HF hospitalization. To ensure that patients with a diagnosis of HF who had never been hospitalized were correctly diagnosed, a further selection was done and only those patients with current prescription of loop-diuretics were selected.

The study used retrospective de-identified data from administrative database and was approved by the local ethics committee of the Hospital del Mar (Parc de Salut Mar) in Barcelona. The three outcome variables of the study were 1-year mortality, and unplanned all cause, and HF hospitalization.

### Assessment of predictors associated with outcomes

We evaluated the independent predictors associated with hospitalization (HF-related and all-cause hospitalization) and all-cause mortality. Predictors assessed were age, sex, comorbidities included in the Charlson Index, previous health care utilization and other comorbidities.

The Charlson Comorbidity Index [16] is generated from the age and morbidity (myocardial infarction, congestive heart failure, peripheral vascular disease, cerebrovascular disease, dementia, chronic obstructive pulmonary disease, connective tissue disease, peptic ulcer disease, diabetes mellitus, moderate to severe chronic kidney disease, hemiplegia, leukemia, malignant lymphoma, solid tumor, liver disease, acquired immune deficiency syndrome) of the patients. This can lead to two problems in multivariable logistic regression. On the one hand, age should not be included again in the model as it would be redundant information. On the other hand, the weight each comorbidity had in the original CCI might not apply nowadays. For instance, ulcer disease has the same weight as heart failure or COPD. For these reasons, in multivariable analysis age and each comorbidity were included individually [17]. This way, it was possible to evaluate the impact of each comorbidity in the population studied.

The codes proposed by Deyo et al were used to build the variables COPD, chronic kidney disease, stroke and dementia [18]. The two categories of diabetes in CCI (with or without organic involvement) were merged in one category and metastases were included in the Cancer category. Ischemic heart disease and cirrhosis were small modifications of the categories “liver disease” and “acute myocardial infarction” found in the original CCI.

Other comorbidities that we analyzed (atrial fibrillation, anemia, cardiac valve diseases and end-stage renal disease) were not included in the CCI. However, we deemed important to include them as they are highly prevalent and have a prognostic value in HF patients. Diagnoses were obtained from the Minimum Basic Dataset for Healthcare Units and from discharge summaries and were defined according to the ICD-9-CM.

The Clinical Classifications Software (CCS) and the Chronic Condition Indicator (ChCI) were used [19] to determine whether a diagnosis was a chronic diagnosis (ChCI) and to eliminate the risk of overlapping diagnosis (CCS). The CCS is a diagnosis categorization scheme based on the ICD-9-CM that aggregates all diagnosis codes into 262 mutually exclusive, clinically homogeneous categories (see S2 File). These groups have been used to construct comorbidity measures to predict the use and costs of hospital services and mortality [20–22].

### Statistical analysis

Analysis of variance was used for comparisons of quantitative variables, which are presented as arithmetic mean (standard deviation); Chi-square was used for qualitative variables. We used multivariable logistic regression to identify independent predictors of worse outcome. The variables were entered in the model one by one and retained when their significance was <0.10. The selection of the best prediction model for identifying patients with the primary outcome was based on the likelihood ratio. Once we identified the best model (for each dependent variable) the area under the receiver operating characteristic (ROC) was calculated [23]. The ROC curves for each outcome were not compared. Statistical analyses were performed using SPSS software, version 18.0. All statistical tests and confidence intervals were constructed with a type I error (alpha) level of 5%, and p-values<0.05 were considered statistically significant.

## Results

The prevalence observed in our study was 1.2% in people older than 15 years and 2.7% in those over 44 years, with a clear increase with ageing: prevalence was 0.3% in the 45–54 year-old group, 0.9% in the 55–64 year-old group, 2.5% between 65–74 and 8.8% in people over 74 years. Table 1 displays the baseline characteristics of the 88,195 patients analyzed in the study.

**Table 1:** Baseline characteristics according to group of diagnosis.

	Total	Never admitted due to HF	Remote HF hospitalization	Recent HF hospitalization	p-value
Cases	88,195	12,407	62,982	12,806	
Age, years, mean ± SD	77.4 ± 12.0	79.9 ± 10.5	76.6 ± 12.4	79.0 ± 10.6	<0.001
Female, n (%)	48,320 (54.8)	8,173 (65,9)	33,026 (52.4)	8,173 (55.6)	<0.001
Number of comorbidities, mean ± SD	5.7 ± 2.0	5.1 ± 2.0	5.7 ± 2.0	6.4 ± 2.0	<0.001
Hypertension, n (%)	85,803 (97.3)	12,407 (100.0)	60,659 (96.3)	12,737 (99.5)	<0.001
Ischemic heart disease, n (%)	42,215 (47.9)	4,375 (35.3)	31,065 (49.3)	6,775 (52.9)	<0.001
Atrial fibrillation, n (%)	41,950 (47.6)	4,464 (36.0)	29,639 (47.1)	7,847 (61.3)	<0.001
Diabetes mellitus, n (%)	37,188 (42.2)	4,259 (34.3)	26,613 (42.3)	6,316 (49.3)	<0.001
Anemia, n (%)	29,429 (33.4)	2,521 (20.3)	21,235 (33.7)	5,673 (44.3)	<0.001
COPD, n (%)	28,612 (32.4)	2,802 (22.6)	20,920 (33.2)	4,890 (38.2)	<0.001
Valve heart disease, n (%)	28,263 (32.0)	1,539 (12.4)	21,074 (33.5)	5,650 (44.1)	<0.001
Chronic kidney disease, n (%)	25,974 (29.5)	2,447 (19.7)	18,207 (28.9)	5,320 (41.5)	<0.001
Depression, n (%)	23,043 (26.1)	3,235 (26.1)	16,202 (25.7)	3,606 (28.2)	<0.001
Cardiac conduction disorders, n (%)	19,865 (22.5)	1,290 (10.4)	14,633 (23.2)	3,942 (30.8)	<0.001
Cancer, n (%)	18,545 (21.0)	2,196 (17.7)	13,506 (21.4)	2,843 (22.2)	<0.001
Stroke, n (%)	16,127 (18.3)	1,776 (14.3)	11,802 (18.7)	2,549 (19.9)	<0.001
Previous acute myocardial infarction, n (%)	13,254 (15.0)	887 (7.1)	10,510 (16.7)	1,857 (14.5)	<0.001
Dementia, n (%)	10,257 (11.6)	1,470 (11.8)	7,179 (11.4)	1,608 (12.6)	<0.001
Cirrhosis, n (%)	2,416 (2.7)	244 (2,0)	1,718 (2.7)	454 (3.5)	<0.001

HF: heart failure; COPD: chronic obstructive pulmonary disease; SD: standard deviation

**Fig 1:**
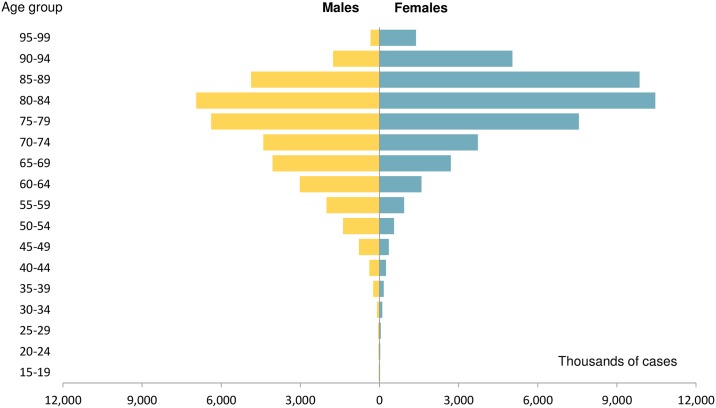
Distribution of heart failure according to age and gender.

Overall, mean age was 77 years, 55% of patients were female and comorbidities were frequent. Fourteen percent of the patients with HF had never been admitted due to HF, and they were more frequently female (2/3 of this group) and old (mean age 79.9 years). The majority of patients (86% of the cohort) had a previous HF hospitalization. Patients with a recent HF hospitalization (15% of population) were somewhat younger but with a much higher burden of comorbidity. Patients with a remote HF admission (71% of the total population) had a comorbidity burden in between the other 2 groups. In patients younger than 74 years, HF was more prevalent in men compared to women. However, women outnumbered men in patients older than 75 years. The number of patients with HF increased as the population aged: 68% of HF patients were 75 years or more and 30% were older than 84 years (Fig 1).

One-year outcome for patients with heart failure is somber. Although 1-year mortality for the whole cohort was 14.3%, patients with a recent HF hospitalization had a 1-year mortality rate of 23.7%, double than those who had never been admitted due to HF. Half of the patients had at least one emergency department (ED) visit and one third of the population was hospitalized during the 1-year follow-up (Table 2).

**Table 2:** One-year outcome and rates of healthcare resource use according to group of diagnosis.

	Total	Never admitted due to HF	Remote HF hospitalization	Recent HF hospitalization	p-value
Mortality rate, n (%)	12,611 (14.3)	1,361 (11.3)	8,188 (13.0)	3,035 (23.7)	<0.001
Patients with an emergency department visit, n (%)	47,096 (53.4)	5,570 (44.9)	33,002 (52.4)	8,554 (66.8)	<0.001
Patients with unplanned HF hospital admission, n (%)	7,725 (8.8)	503 (4.1)	4,369 (6.9)	2,853 (22.3)	<0.001
Patients with unplanned all-cause hospital admission, n (%)	27,164 (30.8)	2,580 (20.8)	18,391 (29.2)	6,121 (47.8)	<0.001
Length of hospitalization, days (per admission), mean ± SD	4.1 ± 10.3	2.4 ± 7.5	3.8 ± 9.7	7.4 ± 13.8	<0.001
Patients with more than one hospital admission, n (%)	10,760 (12.2)	794 (6.4)	6,991 (11.1)	2,907 (22.7)	<0.001
Patients with more than 1 emergency department visit, n (%)	26,634 (30.2)	2,816 (22.7)	18,328 (29.1)	5,532 (43.2)	<0.001
Out-patient specialist contact (per patient)	5.0	3.9	5.1	5.7	<0.001
Primary care contact (per patient)	22.4	21.6	21.6	27.1	<0.001
Patients with use of skilled nursing facility, n (%)	11,377 (12.9)	1,241 (10.0)	7,495 (11.9)	2,650 (20.7)	<0.001

HF: heart failure; SD: standard deviation

Table 3 show the multivariate analysis of factors associated to all-cause and HF hospitalizations and all-cause mortality. The area under the ROC curve for identifying patients with all-cause hospitalization and HF-hospitalization was 0.749 (95% interval confidence (IC) 0.746–0.753) and 0.764 (95% IC 0.758–0.769), respectively, and 0.766 (95% IC 0.762–0.771) for all-cause death.

**Table 3:** Multivariate analysis of factors linked to all-cause and HF hospitalization and all-cause death. Variables with non-statistically significant results are not shown.

Variables	HF hospitalization OR (95% CI)	All-cause hospitalization OR (95% CI)	All-cause mortality OR (95% CI)
**Age (per year)**	1.02 (1.02–1.02)	1.02 (1.02–1.02)	1.07 (1.07–1.07)
**Gender**			
Male (reference)	1	1	1
Female	1.13 (1.07–1.19)	0.91 (0.88–0.94)	0.71 (0.68–0.74)
**Group of HF**			
Remote HF admission (reference)	1	1	1
Primary care setting diagnosis	1.31 (1.19–1.45)	1.18 (1.12–1.24)	-
Recent HF admission	3.42 (3.05–3.83)	1.38 (1.29–1.48)	1.27 (1.17–1.38)
**Presence of any comorbidity**	1.31 (1.29–1.33)	1.42 (1.41–1.44)	1.19 (1.17–1.20)
**Morbidity**			
Ischemic heart disease	1.19 (1.13–1.25)	1.07 (1.03–1.10)	-
Atrial fibrillation	1.42 (1.34–1.49)	1.18 (1.15–1.22)	1.16 (1.11–1.21)
Diabetes mellitus	1.29 (1.23–1.36)	1.12 (1.09–1.16)	1.07 (1.02–1.11)
Anemia	0.91 (0.87–0.96)	0.95 (0.92–0.99)	1.11 (1.07–1.16)
COPD	-	1.06 (1.02–1.10)	-
Valve heart disease	1.33 (1.27–1.40)	1.05 (1.02–1.09)	-
Chronic kidney disease	1.28 (1.21–1.35)	1.04 (1.00–1.07)	1.13 (1.09–1.19)
ESRD-Dialysis	0.40 (0.32–0.51)	-	1.46 (1.26–1.70)
Cardiac conduction disorders	1.22 (1.15–1.28)	-	1.07 (1.02–1.12)
Cancer	0.76 (0.72–0.81)	0.84 (0.81–0.88)	1.16 (1.11–1.22)
Stroke	0.89 (0.84–0.95)	-	1.12 (1.07–1.18)
Dementia	0.65 (0.60–0.70)	0.81 (0.77–0.85)	1.43 (1.36–1.51)
Cirrhosis	0.86 (0.75–0.99)	-	1.58 (1.42–1.77)
**Number of hospitalization during 2012**			
None (reference)	1	1	1
1	0.97 (0.90–1.05)	1.26 (1.20–1.32)	1.15 (1.08–1.22)
2	0.97 (0.88–1.07)	1.38 (1.29–1.47)	1.21 (1.11–1.31)
3	0.96 (0.84–1.10)	1.54 (1.40–1.69)	1.41 (1.27–1.58)
>3	1.21 (1.05–1.40)	2.06 (1.84–2.31)	1.75 (1.54–1.98)
**Number of ED visits during 2012**			
None (reference)	1	1	1
1–2	1.30 (1.21–1.40)	1.43 (1.37–1.50)	1.14 (1.08–1.21)
3–5	1.49 (1.36–1.64)	1.80 (1.70–1.90)	1.32 (1.22–1.42)
>5	1.53 (1.35–1.73)	2.29 (2.10–2.49)	1.55 (1.39–1.71)
Skilled nursing facilities use during 2012	0.69 (0.64–0.75)	0.76 (0.72–0.81)	1.68 (1.59–1.78)

OR: Odds ratio; CI: confidence interval; COPD: Chronic obstructive pulmonary disease; ED: emergency department; ROC: receiver operating characteristic curve; ESRD: End-stage renal disease

## Discussion

In this study, we have shown that at a population level, 68% of HF patients were 75 years or more and comorbidities were frequent. One-year all-cause and HF hospitalization and all-cause mortality were high. Baseline characteristics and outcome were remarkably different depending on the HF population studied. Some comorbidities were associated with both all-cause and HF hospitalizations. Moreover, the presence of diabetes mellitus, atrial fibrillation and chronic kidney disease were independently associated with all-cause and HF hospitalization and with mortality. An all-cause hospitalization or ED visit the previous year was also associated with all-cause and HF-specific hospitalization and mortality.

### Demographics of heart failure patients at a population level

Although in this study HF prevalence was much lower than the one observed in other studies [24,25], it approached values observed by other authors [26]. The lower prevalence we found as compared with some reports could be caused by lack of capture of some patients with mild HF who were not under loop-diuretic. Another explanation could be that at variance with other studies [24] where the denominator in computing prevalence was the number of patients who attended the primary care, internal medicine or cardiology clinics, in our case it was the whole population. Accordingly, application of these two different criteria could explain some discrepancies in HF prevalence. Patients included in this analysis were old (mean age of 77 years), more likely to be female and had a high burden of comorbidity (mean of 5.7 comorbidities per patient). At a population level, 68% of HF patients were 75 years or more and 30% were older than 84 years. Yet, elderly patients have been under-represented in clinical trials. Focusing in the youngest patients leaves the vast majority of HF patients without evidence to guide clinical decision making in this group of patients [27] so effort should be made to increase clinical research in the elderly. Patients with a recent HF admission are high-risk patients, with higher mortality and hospitalization rate and higher 1-year expenditure compared to the other groups [3]. Therefore, many studies have been focused on this group of patients. However, focusing on this group has two main problems. First, at a population level, this group only represents 15% of HF patients. Second, it could lead to under-appreciate the risk of adverse outcome in patients considered to be more stable (those with remote hospitalization or never hospitalized), who nonetheless have frequent events during follow-up. Finally, our study showed a marked difference in the comorbidity burden between the 3 groups of HF diagnosis, which can explain some of the discrepancies seen in different HF studies. An analysis from the MAGGIC program that included individual data on 39,372 patients with HF from 30 cohort studies (six randomized clinical trials and 24 observational registries) showed that patients included in the program had a high cardiovascular comorbidity burden but that other comorbidities were less frequent [28]. Patients included in randomized controlled trials [11,14,29,30] or followed-up in cardiology clinics [31,32] tended to be younger and had a lower prevalence of comorbidities compared to other clinical scenarios. In contrast, patients included after a HF admission had the worst clinical profile, which was similar to that of our population [1,33,34]. In our study, frequency of comorbid conditions in the primary care setting HF diagnosis group (never hospitalized) was similar to other studies done at a community level [5,24,31,35–37]. Similarly to our results Koudstaal et al showed in a population-based linked electronic health record cohort that the burden of comorbidities was different in patients recorded in primary care, acute hospital admissions or both, and that the lower burden of comorbidities was found in patients who had never been hospitalized [38].

### Outcomes

Similarly to what happen with baseline characteristics, long-term outcomes vary depending on the setting the study was carried out and the length of follow-up. Whereas 1-year mortality was as low as 7% in a study of ambulatory HF patients followed-up in cardiology clinics [31], it rose to 17% in acutely decompensated HF patients [31]. At 2 to 3-year follow-up, mortality was 17% to 40% [14,29,39] and after more than 6 years of follow-up, only 22–44% of the patients were alive [34,38]. Hospitalizations are detrimental in HF patients [5]. HF hospitalization was 10–20% in ambulatory patients at 2 year follow-up [35], but rose to 25% at 1-year follow-up in acutely decompensated HF patients [31], which is similar to our results. All-cause hospitalizations are frequent in patients with HF, yet most of the studies have only focused on HF hospitalization. In our study, only 8.8% of patients had a HF hospitalization at 1-year follow-up whereas 30.8% had an all-cause hospitalization (i.e. 22% of patients had a non-HF hospitalization). This is similar to other studies that have shown that all-cause hospitalization in HF patients can be as high as 58% during 1-year follow-up and are associated with worse outcome [6–10,40].

### Factors associated with outcomes

Age and comorbidities are independently associated with all-cause and HF hospitalization [2,35]. In 2014, non-cardiovascular disease was the underlying cause of death for 35% of HF-related deaths, which was an increase from 29% in 2000 [41]. Hence, the identification of comorbidities associated with hospitalizations and mortality would allow us to rapidly identify high-risk patients in order to improve their prognosis. However, few studies have analyzed whether the same comorbidities can predict both HF-specific and all-cause hospitalization or mortality. In our study, the presence of diabetes mellitus, atrial fibrillation or chronic kidney disease was independently associated with both HF and all-cause hospitalization and with mortality in HF patients. The presence of ischemic heart disease and valve heart disease was associated with all-cause and HF-specific hospitalization but not with mortality. Though the importance of some of the comorbidities to predict hospitalization have been described in other publications (diabetes mellitus [38,42]), not all the studies have found the same comorbidities to be predictive of hospitalization (atrial fibrillation [14,38]). This might be due to the fact that the populations analyzed were different, the follow-up was shorter or longer or the end-point was a combination of hospitalization and death [5,12,37,38,43,44]. The presence of ESRD-dialysis, dementia, cirrhosis, cancer and stroke was associated with increased risk of mortality but lower risk for hospitalization. Although that might be unexpected, elderly patients with these comorbidities are often considered to be too sick to be admitted in acute-care hospitals. Instead, they might be admitted in skilled nursing facilities or hospice or treated at home by palliative care teams. Thus, these diseases are associated with low hospital admission but high mortality. In ESRD-dialysis, the fact that the volume overload can be controlled by adjusting the parameters during dialysis adds to the former explanation.

Prior cardiovascular [37], all-cause [43,45] or HF hospitalization (usually defined as a HF admission the previous year) [12,13,46,47] have been associated with death or hospitalization in patients with HF [30,38] and a history of HF is also associated with an increased risk of hospitalization [38,47]. Repeated hospitalizations are frequent in HF patients and associated with increased mortality [14,48,49]. Patients with 1 or more HF hospitalizations had worse baseline characteristics [14] and were at higher risk of subsequent hospitalization or death soon after a hospitalization [47,50]. Similarly, in our study we saw that patients with a recent hospitalization were at risk of mortality or hospitalization, and this risk was particularly high for HF hospitalization (OR 3.4). Interestingly, patients with a diagnosis of heart failure after a remote (>1 year) heart failure admission had a profile and outcome that was between the recent HF hospitalization and patients never hospitalized, likely reflecting a survival bias, as those who died after a recent hospitalization did not enter the remote hospitalization group later on. As the number of all-cause hospitalization during 2012 increased, so did the risk for all-cause hospitalization and mortality. The risk for HF hospitalization significantly increased after more than 3 hospitalizations. Finally, an ED visit was associated with an increase on all-cause and HF hospitalization and all-cause death in our study, and this association was stronger as the number of ED visits increased. Interestingly, this increased risk was independent of the problem that motivated the ED visit, and many patients might have sought care for a variety of reasons unrelated to HF. Information on the role of an ED visit in the outcome of patients with HF is sparse. Au et al showed that after a HF admission, 71.5% patients who had unplanned readmission or death within 30 days of index discharge had had an ED visit at least once in the prior 6 months, a higher proportion than those who were not readmitted [43]. On the other hand, after an ED visit due to HF, lower rates of HF admission were associated with higher rates of repeat ED visits or hospitalizations after previous ED discharge [51] and the lack of prompt follow-up post-ED visit for HF is associated with a higher risk for hospitalization, ED visit and mortality [52]. Therefore, these studies suggest that a more aggressive approach with close follow-up or hospital admission is warranted in this high-risk population.

### Limitations

Although the use of administrative data to identify HF patients can lead to HF misdiagnosis, previous studies and a recent meta-analysis have shown that most of HF diagnoses in administrative databases do correspond to true HF cases. However, the sensitivity of this approach is low, and about one-quarter of HF cases are not captured [53,54]. Unfortunately, ejection fraction was not documented in the administrative database and, thus, it could not be analyzed. Though there is controversy on the effect of ejection fraction in outcomes, different studies [6,34,46] suggest that ejection fraction has little influence on hospitalization or death risks. Although some outcomes can vary according to ejection fraction (different rate of HF-and all-cause hospitalization, for instance), all HF patients experience high rate of adverse outcomes in follow-up irrespective of ejection fraction [10].

## Conclusions

Baseline characteristics and outcome are remarkably different depending on the HF population studied, but the global outcomes of HF are grim. Therefore, it is of paramount importance to identify which factors are associated with mortality and hospitalization in HF patients so resources can be funneled to this high-risk population in order to improve outcomes. The presence of diabetes mellitus, atrial fibrillation and chronic kidney disease were independently associated with all-cause and HF hospitalization and all-cause mortality. An all-cause hospitalization or ED visit the previous year was also associated with all-cause and HF hospitalization and mortality.

## Supporting information

S1 File International classification of diseases, ninth revision, clinical modification (ICD-9-CM) codes used for the diagnosis of heart failure. (PDF)

S2 File Clinical Classifications Software (CCS). The CCS is a diagnosis categorization scheme based on the ICD-9-CM that aggregates all diagnosis codes into 262 mutually exclusive, clinically homogeneous categories**.**
(PDF)

## Abbreviations

ED: Emergency Department
HF: Heart Failure
ICD-9-CM: International Classification of Diseases, Ninth Revision, Clinical Modification
ROC curve: Receiver Operating Characteristic curve
