# Supplementary material for: Real world heart failure epidemiology and outcome: A population-based analysis of 88,195 patients
Source: PLoS One. 2017 Feb 24;12(2):e0172745. doi: 10.1371/journal.pone.0172745 (PMC5325273; doi:10.1371/journal.pone.0172745)
Supplement: S1 File — (PDF) [file pone.0172745.s001.pdf]

S1 File. International classification of diseases, Ninth revision, clinical modification (ICD-9-CM) codes used for the diagnosis of heart failure.

- 39891 Rheumatic heart failure
- 40201 Malignant hypertensive heart disease with heart failure
- 40211 Benign hypertensive heart disease with heart failure
- 40291 Unspecified hypertensive heart disease with heart failure
- 40401 Hypertensive heart and chronic kidney disease, malignant, with heart failure and with chronic kidney disease stage I through stage IV, or unspecified
- 40403 Hypertensive heart and chronic kidney disease, malignant, with heart failure and with chronic kidney disease stage V or end stage renal disease
- 40411 Hypertensive heart and chronic kidney disease, benign, with heart failure and with chronic kidney disease stage I through stage IV, or unspecified
- 40413 Hypertensive heart and chronic kidney disease, benign, with heart failure and chronic kidney disease stage V or end stage renal disease
- 40491 Hypertensive heart and chronic kidney disease, unspecified, with heart failure and with chronic kidney disease stage I through stage IV, or unspecified
- 40493 Hypertensive heart and chronic kidney disease, unspecified, with heart failure and chronic kidney disease stage V or end stage renal disease
- 4280 Congestive heart failure, unspecified
- 4281 Left heart failure
- 42820 Systolic heart failure, unspecified
- 42821 Acute systolic heart failure
- 42822 Chronic systolic heart failure
- 42823 Acute on chronic systolic heart failure
- 42830 Diastolic heart failure, unspecified

- 42831 Acute diastolic heart failure
- 42832 Chronic diastolic heart failure
- 42833 Acute on chronic diastolic heart failure
- 42840 Combined systolic and diastolic heart failure, unspecified
- 42841 Acute combined systolic and diastolic heart failure
- 42842 Chronic combined systolic and diastolic heart failure
- 42843 Acute on chronic combined systolic and diastolic heart failure
- 4289 Heart failure, unspecified
