# Supplementary material for: Real world heart failure epidemiology and outcome: A population-based analysis of 88,195 patients
Source: PLoS One. 2017 Feb 24;12(2):e0172745. doi: 10.1371/journal.pone.0172745 (PMC5325273; doi:10.1371/journal.pone.0172745)
Supplement: S2 File — The CCS is a diagnosis categorization scheme based on the ICD-9-CM that aggregates all diagnosis codes into 262 mutually exclusive, clinically homogeneous categories. (PDF) [file pone.0172745.s002.pdf]

S2 File. Clinical Classifications Software (CCS). The CCS is a diagnosis categorization scheme based on the ICD-9-CM that aggregates all diagnosis codes into 262 mutually exclusive, clinically homogeneous categories.

Ischemic heart disease

410.xx	Acute myocardial infarction
411.xx	Other acute and subacute forms of ischemic heart disease
412	Old myocardial infarction
413.xx	Angina pectoris
414.xx	Coronary atherosclerosis
996.03	Mechanical complication due to coronary bypass graft
V45.81	Aortocoronary bypass status
V45.82	Percutaneous transluminal coronary angioplasty status

Atrial fibrillation

427.31	Atrial fibrillation
427.32	Atrial flutter

Diabetes mellitus

250.xx	Diabetes mellitus

Anemia

280.xx	Iron deficiency anemia
281.xx	Other deficiency anemia
282.xx	Hereditary hemolytic anemia
283.xx	Acquired hemolytic anemia
284.xx	Aplastic anemia
285.xx	Other Anemia

COPD

491.2x	Obstructive chronic bronchitis
491.8	Other chronic bronchitis
491.9	Unspecified chronic bronchitis
492.x	Emphysema
493.2x	Chronic obstructive asthma
494.x	Bronchiectasis
496	Chronic airway obstruction, not elsewhere classified

Valve heart disease

394.x	Mitral stenosis

395.x	Rheumatic aortic stenosis
396.x	Mitral valve stenosis and aortic valve stenosis
397.x	Diseases of tricuspid valve
424.xx	Valve and endocardium disorders
7852	Undiagnosed cardiac murmurs
7853	Other abnormal heart sounds
V422	Heart valve replaced by transplant
V433	Heart valve replaced by other means

Chronic kidney disease

40301	Hypertensive chronic kidney disease, malignant, with chronic kidney disease stage V or end stage renal disease
40311	Hypertensive chronic kidney disease, benign, with chronic kidney disease stage V or end stage renal disease
40391	Hypertensive chronic kidney disease, unspecified, with chronic kidney disease stage V or end stage renal disease
40402	Hypertensive heart and chronic kidney disease, malignant, without heart failure and with chronic kidney disease stage V or end stage renal disease
40403	Hypertensive heart and chronic kidney disease, malignant, with heart failure and with chronic kidney disease stage V or end stage renal disease
40412	Hypertensive heart and chronic kidney disease, benign, without heart failure and with chronic kidney disease stage V or end stage renal disease
40413	Hypertensive heart and chronic kidney disease, benign, with heart failure and chronic kidney disease stage V or end stage renal disease
40492	Hypertensive heart and chronic kidney disease, unspecified, without heart failure and with chronic kidney disease stage V or end stage renal disease
40493	Hypertensive heart and chronic kidney disease, unspecified, with heart failure and chronic kidney disease stage V or end stage renal disease
45821	Hypotension of hemodialysis
582.xx	Chronic glomerulonephritis
5830	Nephritis and nephropathy, not specified as acute or chronic, with lesion of proliferative glomerulonephritis
5831	Nephritis and nephropathy, not specified as acute or chronic, with lesion of membranous glomerulonephritis
5832	Nephritis and nephropathy, not specified as acute or chronic, with lesion of membranoproliferative glomerulonephritis
5834	Nephritis and nephropathy, not specified as acute or chronic, with lesion of rapidly progressive glomerulonephritis
5836	Nephritis and nephropathy, not specified as acute or chronic, with lesion of renal cortical necrosis
5837	Nephritis and nephropathy, not specified as acute or chronic, with lesion of renal medullary necrosis
585.x	Chronic kidney disease
586	Renal failure, unspecified
5880	Renal osteodystrophy
7925	Cloudy (hemodialysis) (peritoneal) dialysis effluent
99681	Complications of transplanted kidney

99673	Other complications due to renal dialysis device, implant, and graft
99656	Mechanical complication due to peritoneal dialysis catheter
V42.0	Kidney replaced by transplant
V45.1x	Renal dialysis status
V56.xx	Encounter for extracorporeal dialysis

Cancer

140.xx	Malignant neoplasm of lip
141.xx	Malignant neoplasm of tongue
142.xx	Malignant neoplasm of salivary gland
143.xx	Malignant neoplasm of gum
144.xx	Malignant neoplasm of floor of mouth
145.xx	Malignant neoplasm of mouth
146.xx	Malignant neoplasm of oropharynx
147.xx	Malignant neoplasm of nasopharynx
148.xx	Malignant neoplasm of hypopharynx
149.xx	Malignant neoplasm of ill-defined sites within the lip and oral cavity
150.xx	Malignant neoplasm of esophagus
151.xx	Malignant neoplasm of stomach
152.xx	Malignant neoplasm of small intestine
153.xx	Malignant neoplasm of colon
154.xx	Malignant neoplasm of rectum, rectosigmoid junction, and anus
155.xx	Malignant neoplasm of liver and intrahepatic bile ducts
156.xx	Malignant neoplasm of biliary tract
157.xx	Malignant neoplasm of pancreas
158.xx	Malignant neoplasm of peritoneum
159.xx	Malignant neoplasm of ill-defined sites within the digestive organs and peritoneum
160.xx	Malignant neoplasm of accessory sinus
161.xx	Malignant neoplasm of larynx
162.xx	Malignant neoplasm of bronchus and lung
163.xx	Malignant neoplasm of pleura
164.xx	Malignant neoplasm of mediastinum
165.xx	Malignant neoplasm of ill-defined sites within the respiratory system
170.xx	Malignant neoplasm of bone and articular cartilage
171.xx	Malignant neoplasm of connective and other soft tissue,
172.xx	Melanoma of skin
173.xx	Malignant neoplasm of skin
174.xx	Malignant neoplasm of breast (female)
175.xx	Malignant neoplasm of male breast
176.xx	Kaposi's sarcoma
179.xx	Malignant neoplasm of uterus
180.xx	Malignant neoplasm of cervix uteri
181.xx	Malignant neoplasm of placenta
182.xx	Malignant neoplasm of corpus uteri

183.xx	Malignant neoplasm of uterine adnexa
184.xx	Malignant neoplasm of female genital organ
185.xx	Malignant neoplasm of prostate
186.xx	Malignant neoplasm of testis
187.xx	Malignant neoplasm of male genital organ,
188.xx	Malignant neoplasm of bladder
189.xx	Malignant neoplasm of urinary organ
190.xx	Malignant neoplasm of eye
191.xx	Malignant neoplasm of brain
192.xx	Malignant neoplasm of nervous system
193.xx	Malignant neoplasm of thyroid gland
194.xx	Malignant neoplasm of endocrine gland
195.xx	Malignant neoplasm of other specified sites
196.xx	Secondary and unspecified malignant neoplasm of lymph nodes
197.xx	Secondary malignant neoplasm of respiratory and digestive organs
198.xx	Secondary malignant neoplasm of other specified sites
199.xx	Other malignant neoplasm without specification of site
200.xx	Limfosarcoma and Reticulosarcoma
201.xx	Hodgkin's disease
202.xx	Nodular lymphoma
203.xx	Multiple myeloma
204.xx	lymphoid leukemia
205.xx	myeloid leukemia,
206.xx	monocytic leukemia
207.xx	Other leukemia
208.xx	Acute leukemia of unspecified cell type
209.0x	Malignant carcinoid tumor of the small intestine,
209.1x	Malignant carcinoid tumor of the large intestine
209.2x	Malignant carcinoid tumor of unknown primary site
209.3x	Malignant poorly differentiated neuroendocrine carcinoma
209.7x	Secondary neuroendocrine tumor
230.xx	Carcinoma in situ of digestive organs
231.xx	Carcinoma in situ of respiratory system
232.xx	Carcinoma in situ of skin
233.xx	Carcinoma in situ of urinary organs
234.xx	Carcinoma in situ, site unspecified

Stroke

094.87	Persistent migraine aura with cerebral infarction
346.6x	Subarachnoid hemorrhage
430	Intracerebral hemorrhage
431	Nontraumatic extradural hemorrhage
432.x	Occlusion and stenosis of basilar artery without mention of cerebral infarction
433.xx	Cerebral thrombosis without mention of cerebral infarction

434.xx	Acute, but ill-defined, cerebrovascular disease
436	Other cerebrovascular disease
437	Cerebral atherosclerosis
4370	Other generalized ischemic cerebrovascular disease
4371	Other ill-defined cerebrovascular disease
4378	Unspecified cerebrovascular disease
4379	late effects of cerebrovascular disease
438.xx	Aphasia
7843	Iatrogenic cerebrovascular infarction or hemorrhage
99702	Persistent migraine aura with cerebral infarction

Dementia

290.xx	Senile psychotic condition
294.1x	Dementia in conditions classified elsewhere
331.0	Alzheimer's disease
331.1x	Frontotemporal dementia
3312	Senile degeneration of brain

Cirrhosis

571.2	Alcoholic cirrhosis of liver
571.5	Cirrhosis of liver without mention of alcohol
